# Prolonged dengue viremia with predominantly ocular manifestations in a lung transplant recipient

**DOI:** 10.1186/s12879-025-11456-7

**Published:** 2025-08-14

**Authors:** Leong Shuen Loo, Michael Zhang, Jane Wells, Noor Ali, Ian Marr

**Affiliations:** 1 Department of Infectious Diseases, The Canberra Hospital, Canberra, ACT Australia; 2 Department of Ophthalmology, The Canberra Hospital, Canberra, ACT Australia; 3 Menzies School of Health, Darwin, Australia

**Keywords:** Dengue, Immunocompromised host, Uveitis, Retinitis, Lung transplant

## Abstract

**Background:**

The clinical manifestations of dengue are well-described, but ocular involvement is being increasingly recognized and alterations to the natural trajectory of disease, including protracted viraemia, have also been recognized in immunocompromised patients. We describe a case of dengue in a lung transplant recipient which manifested predominantly as ocular symptoms following short-lived systemic features, and with protracted viraemia with delayed IgM to IgG seroconversion.

**Case presentation:**

A 40-year-old woman who had a bilateral lung transplant 15 years prior for cystic fibrosis presented with headaches, bilateral scotomata and rapidly deteriorating visual acuity. She had recently travelled to Bali where she experienced a short-lived febrile illness featuring a retro-orbital headache and generalized arthralgia. Ocular examination demonstrated bilateral cystoid macular oedema, retinitis and retinal vasculitis. Dengue serotype 1 RNA was detected in serum, urine and aqueous humour samples. Dengue serology showed positive NS1 and IgM, and negative IgG. She had presented 14 days after the initial febrile illness began. High-dose prednisolone was commenced for the macular oedema, but this was stopped when low-grade fevers and arthralgias developed. Her immunosuppression was reduced, with clinical improvement to visual acuity and macular oedema observed, but high-dose prednisolone was recommenced at day 36 of illness due to new retinal haemorrhages, this time well-tolerated, with subsequent dose tapering. Viraemia was protracted, clearing at day 69, and IgG seroconversion was noted on day 319. Her vision improved sufficiently to allow driving and return to part-time work, though she continues to experience persisting symptomatic right-sided macular oedema.

**Conclusions:**

This was an unusual presentation of dengue involving an immune-privileged site in an immunocompromised host. The competing priorities of facilitating immune-mediated clearance of viraemia versus controlling ocular inflammation posed a significant therapeutic challenge. A greater understanding of the pathophysiology of dengue eye disease, including virus-mediated and immune-mediated factors, as well as the development of therapeutic options, is critically required.

## Background

The cardinal clinical manifestations and natural history of dengue are well-described [1], but ocular manifestations such as uveitis are becoming increasingly well-recognized [2], and alterations to the time course of viraemia have been observed in immunocompromised patients [3, 4]. We describe an unusual presentation of dengue in a lung transplant recipient that manifested predominantly with ocular symptoms that followed a short-lived systemic syndrome, and with protracted viraemia and delayed IgM to IgG seroconversion.

## Case report

**Fig. 1:**
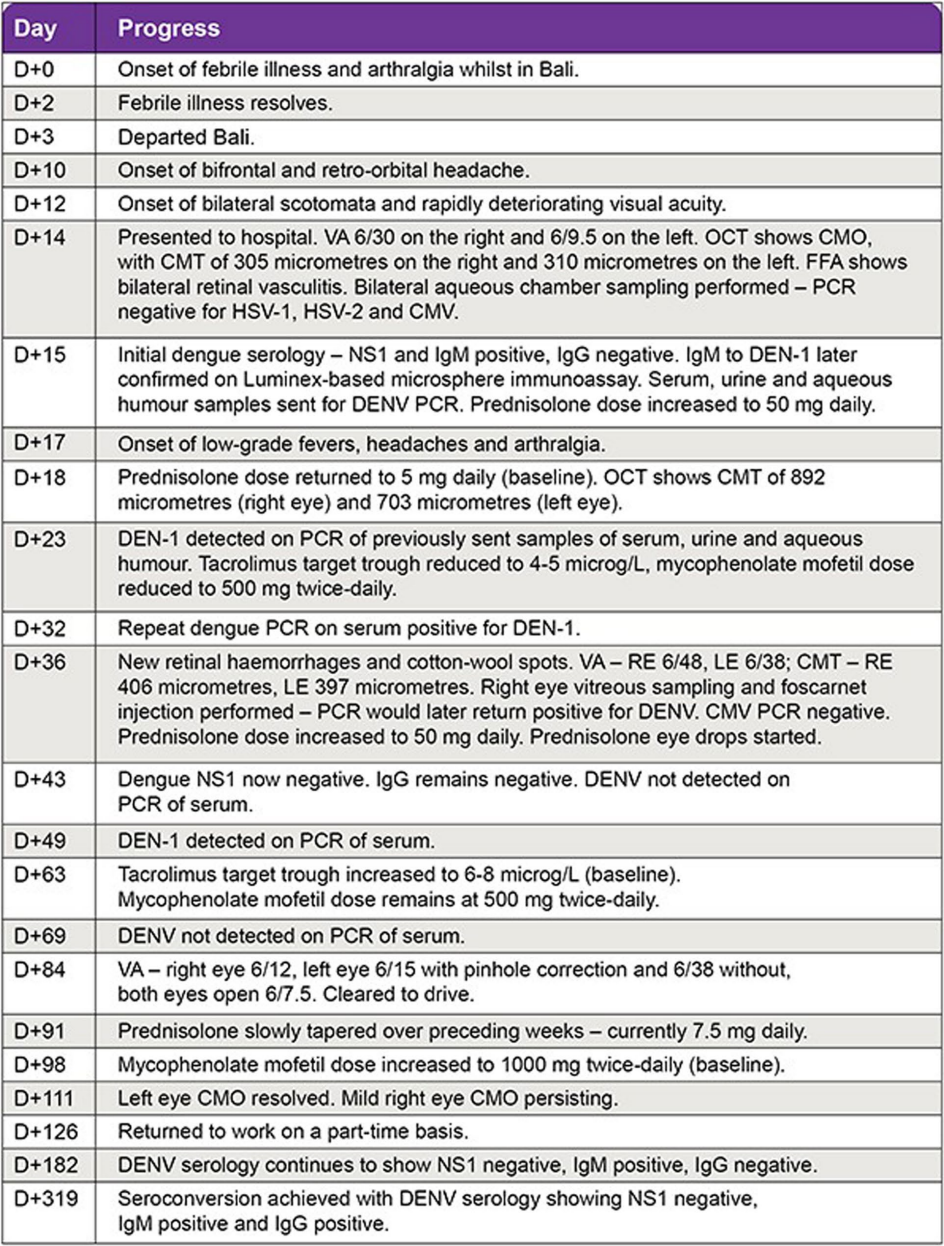
Timeline of key clinical and ocular findings, investigation results and treatment given

A 40-year-old Caucasian woman was referred to the ophthalmology clinic by her optometrist with a two-day history of bilateral scotomata and rapidly deteriorating visual acuity, alongside a four-day history of bifrontal and retro-orbital headache. She further reported mild photophobia and orbital fullness, but denied diplopia, flashes, amaurosis, pain with eye movements, neck stiffness and phonophobia. She had no history of prior ocular pathology but had had a bilateral lung transplant for cystic fibrosis 15 years prior and was stable on tacrolimus 1.5 mg twice-daily (target trough level 6–8 microg/L), mycophenolate mofetil 1000 mg twice-daily and prednisolone 5 mg daily. She had never experienced rejection episodes but had one episode of cytomegalovirus (CMV) colitis seven years prior. She had recently returned from a two-week trip to Bali, Indonesia. While overseas, she experienced a febrile illness featuring a retro-orbital headache and generalized arthralgia that self-resolved after two days. She departed Bali three days after this febrile illness started (D + 3, where D + 0 is the first day of the febrile illness). The presenting headache started ten days after the febrile illness began (D + 10), the visual symptoms on D + 12, and hospital presentation was on D + 14. She had not travelled elsewhere recently and had no sick contacts. A timeline of key events is shown in Fig. 1.

**Fig. 2:**
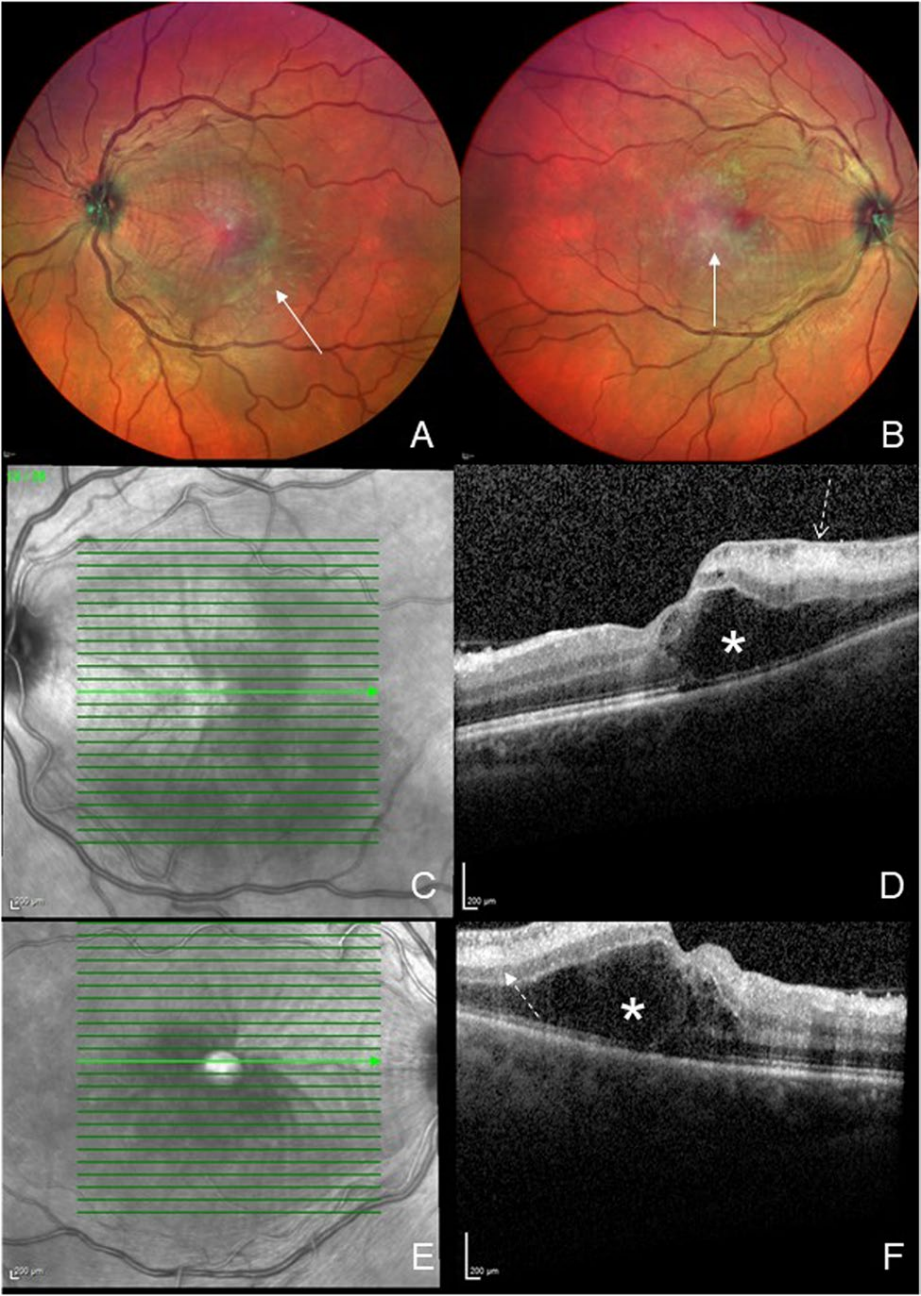
Ocular imaging of the eye obtained on D+14 of illness. **A** and **B**: Colour fundus photography of the left and right eye respectively showing scattered macular retinitis (arrows). **C**-**F**: Ocular coherence tomography of the left (**C** and **D**) and right (**E** and **F**) eyes through the fovea showing macular oedema (asterisks) and retinitis (dashed arrows)

There was no nausea, vomiting, abdominal pain, myalgia, arthralgia or rash at the time of admission. Vital signs, blood counts, haematocrit, electrolytes, renal and liver function tests were normal. Visual acuity (VA) was 6/30 on the right and 6/9.5 on the left on admission, which worsened to counting fingers on the right and 6/60 on the left the next day. Fundoscopy (Fig 2 A-B) showed bilateral cystoid macular oedema (CMO), nasal optic disc swelling, intra-retinal haemorrhages and chorioretinal atrophy. Ocular coherence tomography (OCT) (Fig 2 C-F) showed bilateral CMO with central macular thickness (CMT) of 305 micrometres on the right, and 310 micrometres on the left. Fundus fluorescein angiography showed bilateral retinal vasculitis (phlebitis more so than arteritis) and non-specific diffuse hyperfluorescent white lesions. Bilateral aqueous humour aspiration followed by intravitreal foscarnet injections were performed to investigate for and pre-emptively treat CMV retinitis given the history of CMV disease, but all samples were negative on polymerase chain reaction (PCR) for CMV, herpes simplex virus 1 and 2. Alphavirus IgM and IgG were sent to investigate for chikungunya, which returned negative.

Dengue virus (DENV) serology performed on D + 15 returned as NS1 positive, IgM positive and IgG negative. Given these results, serum, urine and the bilateral aqueous humour samples were sent to a reference laboratory for DENV PCR and confirmatory flavivirus serology. Prednisolone 50 mg daily was commenced on D + 15 for the ocular inflammation, but low-grade fevers, headaches and arthralgias resembling the initial illness occurred, and the dose was returned to 5 mg daily on D + 18. CMT peaked at this time (right eye (RE) 892 micrometres, left eye (LE) 703 micrometres). The PCR results of serum, urine and aqueous humour returned on D + 23; all were positive for DENV serotype 1 (DEN-1), and a Luminex-based microsphere immunoassay [5] detected IgM to DEN-1. To aid clearance of DENV, tacrolimus dosing was titrated to a lower target trough level of 4–5 microg/L, and mycophenolate mofetil was reduced to 500 mg twice-daily, without complications. VA and CMO improved on serial eye examinations. On D + 36, while VA and CMO continued to show improvement (RE VA 6/48 and CMT 406 micrometres, LE VA 6/38, CMT 397 micrometres), new retinal haemorrhages with increased intraocular pressure were noted on examination, prompting reinitiation of prednisolone 50 mg, this time without adverse effects. PCR of vitreous fluid from the right eye at this time was negative for CMV, but positive for DENV. VA and CMO continued to improve, and tapering of the prednisolone dose was commenced. Further serum samples were sent for DENV PCR on D + 32, D + 43, D + 49, D + 69 and D + 89; DEN-1 was detected on the samples from D + 32 and D + 49, but not on the samples from D + 43, D + 69 and D + 89. The patient was able to resume driving on D + 84, VA being 6/12 on the right, 6/15 with pinhole correction and 6/38 without on the left and 6/7.5 with both eyes open at that time. The prednisolone dose was 7.5 mg daily by D + 91. Tacrolimus and mycophenolate mofetil doses were returned to their original prescribed regimen (prior to dengue onset) by D + 98. The left eye CMO resolved by D + 111. The patient resumed part-time work on D + 126 whilst continuing prednisolone 7.5 mg daily and prednisolone eye drops for fluctuating symptomatic right-sided CMO, manifesting as scotoma and blurred vision. DENV serology performed on D + 182 showed NS1 negative, IgM positive and IgG negative, but subsequent serology on D + 319 demonstrated seroconversion with NS1 negative, IgM positive and IgG positive.

## Discussion

Reported manifestations of dengue eye disease have included maculopathy, retinal vasculitis, retinal haemorrhage, subconjunctival haemorrhage and anterior uveitis, the former three being observed in our patient. Most cases experience spontaneous improvement, though long-term visual sequelae has also been observed [1, 2, 6, 7]. Where maculopathy is present, the pattern observed on OCT has been used to inform prognosis, with the Type 2 pattern (cystoid macular oedema, the case for our patient) being generally associated with a good visual prognosis [8]. Key differentials to suspected dengue eye disease include chikungunya and Zika virus infections [9]; in our case the former was ruled out on serology and the latter not investigated for as we returned a positive DENV NS1 result within a day of her presentation. Infection with DEN-1, the serotype in our patient, also appears to be associated with an increased risk of ocular disease [10]. The mechanisms of ocular disease in dengue are not well understood, with both viral and immune-mediated factors postulated. DENV has demonstrated a direct cytopathic effect on retinal pigment epithelial cells in vitro [11], but visual symptoms often onset after the sixth or seventh day of illness [6, 7], when viraemia is declining [1, 12]. In our patient, DENV was detected by PCR in ocular and serum samples over a month after initial infection. To our knowledge, detection of DENV from ocular samples has not been previously described. While the initial increase in steroid dose was followed by a return of a dengue-like clinical syndrome, the second commencement of high-dose prednisolone did not, and eye symptoms did not worsen in either instance. The background immunosuppression would have further attenuated an immune response.

A competing priority in our patient was the prolonged viraemia and concerns that high-dose steroids could impair clearance of viraemia and facilitate worsening systemic infection, balancing this against the need to control the ocular inflammation. Serum PCR for DENV generally becomes negative within one to two weeks [1, 12], though prolonged viraemia has been observed in haematopoietic stem cell transplant [3] and renal transplant [4] recipients, and even immunocompetent patients [13]. We acknowledge that detection of DEN-1 on PCR does not confirm replicating competent virus, and viral cultures were not done. It is further noted that negative viral cultures with ongoing positive PCR in blood have been observed [4, 13]. In our patient, we postulate that the initial predominant mechanism of the observed retinitis and vasculitis could be due to a direct viral cytopathic effect on endothelial and retinal cells, with impaired cellular immunity and the immune-privileged nature of the eye accounting for the ocular without systemic symptoms. Reduction of immunosuppression could have enabled a degree of immune recovery that controlled viral replication but allowed immune-mediated inflammation to predominate, resulting in the clinical findings described at D + 36.

## Conclusion

We present a case of prolonged dengue viraemia in a lung transplant recipient, manifesting primarily with ocular symptoms. The competing priorities of facilitating immune clearance of viraemia and attenuating immune-mediated damage complicated management. With the increasing incidence and geographical distribution of dengue [1], the increasing prevalence of immunosuppression for various diseases and an ongoing absence of antiviral options for dengue [14], a better understanding of the pathophysiology of dengue and development of therapeutic options are critically needed.

## Data Availability

No datasets were generated or analysed during the current study.

## Abbreviations

CMV: cytomegalovirus
CMO: cystoid macular oedema
OCT: ocular coherence tomography
CMT: central macular thickness
RE: right eye
LE: left eye
PCR: polymerase chain reaction
DENV: dengue virus
DEN-1: dengue virus serotype 1
